# Two new *Geranomyia* Haliday (Diptera, Limoniidae) crane flies from Mount Jiulong in China, with an updated key to Chinese species

**DOI:** 10.3897/zookeys.953.49557

**Published:** 2020-07-27

**Authors:** Xingyang Qian, Xiao Zhang

**Affiliations:** 1 Key Lab of Integrated Crop Pest Management of Shandong Province, College of Plant Health and Medicine, Qingdao Agricultural University, Qingdao 266109, China Qingdao Agricultural University Qingdao China

**Keywords:** crane fly, Limoniinae, Limoniini, classification, new species, Zhejiang

## Abstract

The genus *Geranomyia* Haliday, 1833 is globally distributed, with 351 known species, of which 26 occur in China. Herein, an overview of the genus *Geranomyia* from Mount Jiulong, Zhejiang, China, is presented. Two new species are described and illustrated. *Geranomyia jiulongensis***sp. nov.** and *G. subablusa***sp. nov.** are distinguished from other *Geranomyia* species by the characters of the thorax and male genitalia. An updated key to the *Geranomyia* of China is presented.

## Introduction

*Geranomyia* Haliday, 1833 is a large genus of 351 described species in the family Limoniidae. It is characterized by the following characters: body small or medium-sized (5–9 mm), flagellum with 12 segments, elongate mouthparts, R_1+2_ present, R_2_ commonly present, R_4_ and R_5_ fused to margin, only two branches of Rs present as longitudinal elements (R_3_ and R_4+5_), two branches of M reaching wing margin, and lobe of gonostylus often with two spines (Alexander 1967a; Haliday 1833; Osten Sacken 1869).

The adults of *Geranomyia* species were often found sucking nectar from flowers (Alexander 1948, 1967b; Zhang et al. 2016). Some phenological studies have shown that adults have a long period of activity; for example, adults of *G. canadensis* (Westwood, 1836) were active from April to September, *G. communis* Osten Sacken, 1860 from May to October, and *G. rostrata* (Say, 1823) from April to September (Young 1978; Young and Gelhaus 2000). The habitats of adult flies have been rather frequently discussed in the literature (Alexander 1916, 1919, 1920, 1928a, 1928b, 1948, 1964, 1970a; Englund 1999; Harrison and Barnard 1972; Knab 1910). *Geranomyia advena* (Alexander, 1954) has been found around seeps and adjacent to riffle habitats in streams on Molokai and Hawaii (Englund 1999). The type of *G. annandalei* Edwards, 1913 was collected on the Plain of Gennesaret, near the Sea of Galilee, where it was found on limestone cliffs overhanging a spring (Alexander 1970b).

The habitats of the immature stages have also been extensively investigated. The larvae of *G. canadensis* was found living on the faces of rock exposures, crawling among algae and diatoms (Alexander and Malloch 1920). Rogers (1927) found the immature stages of *G. rostrata* living in wet moss, among the thalli of liverworts and in mats of filamentous algae on wet rocks and shaded seepage areas. Bangerter (1929) found the larvae of *G. caloptera* Mik, 1867 living among saturated mosses on wet banks of streams. The immature stages of *G. diversa* Osten Sacken, 1860 was found in and beneath thick mats of dripping algae on wet, shaded cliffs (Rogers 1930). *Geranomyia argentifera* de Meijere, 1911 and *G. fletcheri* Edwards, 1911 have habitats that are generally similar to the above-mentioned species (Alexander1931).

Twenty-six species of *Geranomyia* have been previously recorded from China (Oosterbroek 2020), of which five were published by Zhang et al. (2016). Since that publication, further new materials of the genus have become available. Mount Jiulong is located in southwestern Zhejiang, China, with a total area of 200 km^2^. The main peak is 1,724 m high, which is the fourth highest peak in Zhejiang. The area includes more than 6 km^2^ of virgin, typically subtropical, broad-leaf forest. Mount Jiulong is reputed to be a “Biological Gene Pool”, with more than 1,340 species of plants and 149 species of vertebrates. This investigation into *Geranomyia* species on Mount Jiulong, Zhejiang, China, was initiated by the authors together with other entomologists from Zhejiang A&F University in July 2019, and four species of *Geranomyia* were found. In this paper, two new species are described and illustrated, and two known Chinese species are also listed. A dichotomous key to the Chinese species of *Geranomyia* is modified from Zhang et al. (2016) and updated with additional diagnostic characters.

## Materials and methods

Specimens for this study were collected on Mount Jiulong, Zhejiang, China, in July 2019 by the authors. Adult crane flies were collected by insect net and at artificial light. Genitalic preparations of males were made by macerating the apical portion of the abdomen in cold 10% NaOH for 12–15 hours. Observations and illustrations were made using a ZEISS Stemi 2000-C stereomicroscope. Photographs were taken with a Canon EOS 77D digital camera through a macro lens. Type specimens of known Chinese species deposited in the National Museum of Natural History, Smithsonian Institution, Washington, DC, USA (USNM), the Natural History Museum, London, UK (NHM) and the Entomological Museum of China Agricultural University, Beijing, China (CAU) were examined. Type specimens of the new species were deposited in the Entomological Museum of Qingdao Agricultural University, Shandong, China (QAU).

The morphological terminology mainly follows McAlpine (1981), and the venation is described after Alexander and Byers (1981). Terminology of the male hypopygium follows Ribeiro (2006). The following abbreviations are used: tg 9 = ninth tergite, tg 10 = tenth tergite, goncx = gonocoxite, c gonst = clasper of gonostylus, l gonst = lobe of gonostylus, aed = aedeagus, pm = paramere, cerc = cercus, hyp vlv = hypogynial valve, mm = millimeter.

## Taxonomy

### Key to Chinese *Geranomyia*

**Table:** 

1	Wing patterned with dark brown stigma only	**2**
–	Wing patterned with many spots besides stigma (Figs 1d, 3d)	**6**
2	Stigma large, covering about 1/2 of distal section of R_1_	**3**
–	Stigma small, covering about 1/3 of distal section of R_1_	**4**
3	Prescutum with three confluent stripes; ovipositor with tip of hypogynial valve near 1/2 way along cercus	***G. contrita* (Alexander, 1937) (Guangdong)**
–	Prescutum without stripes; ovipositor with tip of hypogynial valve near 3/4 way along cercus	***G. nigra* Zhang, Zhang & Yang, 2016 (Gansu, Sichuan, Zhejiang, Guanxi, Yunnan, Taiwan)**
4	Wing with basal section of CuA_1_ at fork of M	***G. nitida* de Meijere, 1911 (Taiwan; Indonesia)**
–	Wing with basal section of CuA_1_ more than 1/3 of its own length before (Figs 1d, 3d) or beyond fork of M	**5**
5	Wing with basal section of CuA_1_ about 3/4 of its length beyond fork of M; lobe of gonostylus with two short spines directly arising from rostral prolongation	***G. argentifera* (Taiwan, Hainan; Indonesia; Philippines)**
–	Wing with basal section of CuA_1_ about 1/3 of its length before fork of M; lobe of gonostylus with two long spines arising from a tubercle on rostral prolongation	***G. gracilispinosa* (Alexander, 1937) (Guangdong; India; Sri Lanka)**
6	Wing with spots in costal region except stigma small and weak; seams along cord, m-m and basal section of M_3_ almost invisible	**7**
–	Wing with spots in costal region large and dark; seams along cord, m-m and basal section of M_3_ conspicuous (Figs 1d, 3d)	**9**
7	Prescutum with two stripes	***G. atrostriata* Edwards, 1921 (Taiwan)**
–	Prescutum with three stripes	**8**
8	Wing with a few distinct spots at base of R	***G. montana* de Meijere, 1911 (Taiwan; Indonesia)**
–	Wing without conspicuous spot at base of R	***G. sparsiguttata* (Alexander, 1937) (Chongqing, Sichuan, Fujian, Yunnan)**
9	Wing with basal section of CuA_1_ more than 1/3 of its own length before fork of M (Figs 1d, 3d)	**10**
–	Wing with basal section of CuA_1_ less than 1/4 of its length before or beyond fork of M	**16**
10	Lobe of gonostylus with two conspicuous tubercles on rostral prolongation (Fig. 2a)	**11**
–	Lobe of gonostylus with one or no tubercle on rostral prolongation (Fig. 4a)	**12**
11	Prescutum with a narrow brown median stripe; lobe of gonostylus small and short, slightly exceeding clasper of gonostylus	***G. radialis* (Alexander, 1930) (Zhejiang, Guangxi, Taiwan; Japan)**
–	Prescutum with three broad, dark-brown stripes (Fig. 1c); lobe of gonostylus large and long, more than twice length of clasper of gonostylus (Fig. 2a)	***G. jiulongensis* sp. nov. (Zhejiang)**
12	Lobe of gonostylus with two long and slender spines, one arising from a large tubercle on rostral prolongation, other one directly arising from rostral prolongation (Fig. 4a)	**13**
–	Lobe of gonostylus not as above	**14**
13	Wing with spot at fork of Rs restricted under Sc, cell r_3_ without spot under R_2_; lobe of gonostylus with two spines at tip and base of rostral prolongation respectively; distal part of paramere finger-shaped, lateral margin serrated or jagged	***G. tenuispinosa* (Alexander, 1929) (Zhejiang, Fujian, Guangdong, Jiangxi)**
–	Wing with spot at fork of Rs covering Sc and reaching costal margin, cell r_3_ with a spot under R_2_ (Fig. 3d); lobe of gonostylus with two spines at tip and middle of rostral prolongation respectively; distal part of paramere trianglar, lateral margin smooth (Fig. 4a)	***G. subablusa* sp. nov. (Zhejiang)**
14	Wing with a large spot at middle area of cell cua_1_	***G. maculata* Zhang, Zhang & Yang, 2016 (Taiwan)**
–	Wing without conspicuous spot at middle area of cell cua_1_	**15**
15	Pleuron of thorax without stripe; spot between first and second large spots in costal region very faint; lobe of gonostylus with rostral prolongation pointed at apex, middle of rostral prolongation with two subequal spines	***G. obesistyla* (Alexander, 1940) (Sichuan)**
–	Pleuron of thorax with an ill-defined longitudinal stripe; spot between first and second large spots in costal region conspicuous; lobe of gonostylus with rostral prolongation blunt, middle of rostral prolongation with two spines, outer spine a little longer than inner spine	***G. suensoniana* (Alexander, 1929) (Zhejiang)**
16	Wing with Sc_1_ ending at about 1/2 to 2/3 of Rs	**17**
–	Wing with Sc_1_ ending at more than 3/4 of Rs	**20**
17	Wing with spots on origin of Rs and fork of Sc confluent in cell C or nearly so	***G. alpestris* (Alexander, 1930) (Taiwan)**
–	Wing with spots on origin of Rs and fork of Sc distinctly separated	**18**
18	Wing with many small dots near M and CuA	***G. pictorum* (Alexander, 1929) (Taiwan; India)**
–	Wing without small dot near M or CuA	**19**
19	Prescutum with three broad longitudinal stripes	***G. baisensis* Zhang, Zhang & Yang, 2016 (Guangxi)**
–	Prescutum without evident markings	***G. spectata* (Alexander, 1937) (Guangdong)**
20	Wing heavily patterned, a large spot throughout wing tip, spot on origin of Rs posteriorly bifurcated	***G. apicifasciata* (Alexander, 1930) (Guangdong, Yunnan, Taiwan)**
–	Wing not as above	**21**
21	Wing without conspicuous spot at base	***G. kiangsiana* (Alexander, 1937) (Jiangxi)**
–	Wing with spot at base	**22**
22	Prescutum with a median longitudinal stripe	***G. unifilosa* (Alexander, 1934) (Taiwan)**
–	Prescutum with three longitudinal stripes	**23**
23	Legs uniformly light brownish	***G. septemnotata* Edwards, 1916 (Taiwan)**
–	Legs pale yellow to brownish yellow, with tibiae and tarsi darker, or femora with tips darker or bases paler	**24**
24	Costal region of wing with a small spot in cell C between second and third large spots	***G. fremida* (Alexander, 1937) (Guangdong)**
–	Costal region of wing without conspicuous spot in cell C between second and third large spots	**25**
25	Lobe of gonostylus with two spines	***G. subradialis* (Alexander, 1937) (Guangdong)**
–	Lobe of gonostylus with one spine	**26**
26	Lobe of gonostylus with rostral prolongation small, a very long and slender spine arising from a tubercle on rostral prolongation	***G. longispina* Zhang, Zhang & Yang, 2016 (Fujian)**
–	Lobe of gonostylus with rostral prolongation long and slender, a long and powerful spine directly arising from rostral prolongation	**27**
27	Male hypopygium with posterior margin of tergite nine deeply and narrowly notched; clasper of gonostylus small, slender, and nearly straight	***G. bifurcula* (Alexander, 1933) (Sichuan)**
–	Male hypopygium with posterior margin of tergite nine emarginate; clasper of gonostylus absent	***G. degenerata* Zhang, Zhang & Yang, 2016 (Guangxi)**


#### Geranomyia jiulongensis sp. nov.

Taxon classificationAnimaliaDipteraLimoniidae

9868E2F5-778E-5DFE-8F1C-650100A87C67

http://zoobank.org/2E46B202-AA91-4C18-9421-6355DE078719

Figures 1 , 2

##### Diagnosis.

Prescutum yellow with three broad, dark-brown longitudinal stripes. Pleuron of thorax yellow, with a broad, dark-brown stripe. Wing with seven large spots on costal region; Sc_1_ ending near fork of Rs, basal section of CuA_1_ nearly its length before fork of M. Lobe of gonostylus large with an arched rostral prolongation armed with two basal spines arising from two tubercles.

##### Description.

**Male.** Body length 5.0–5.3 mm, wing length 5.3–5.5 mm, mouthparts length 2.2–2.3 mm.

**Figure 1.**
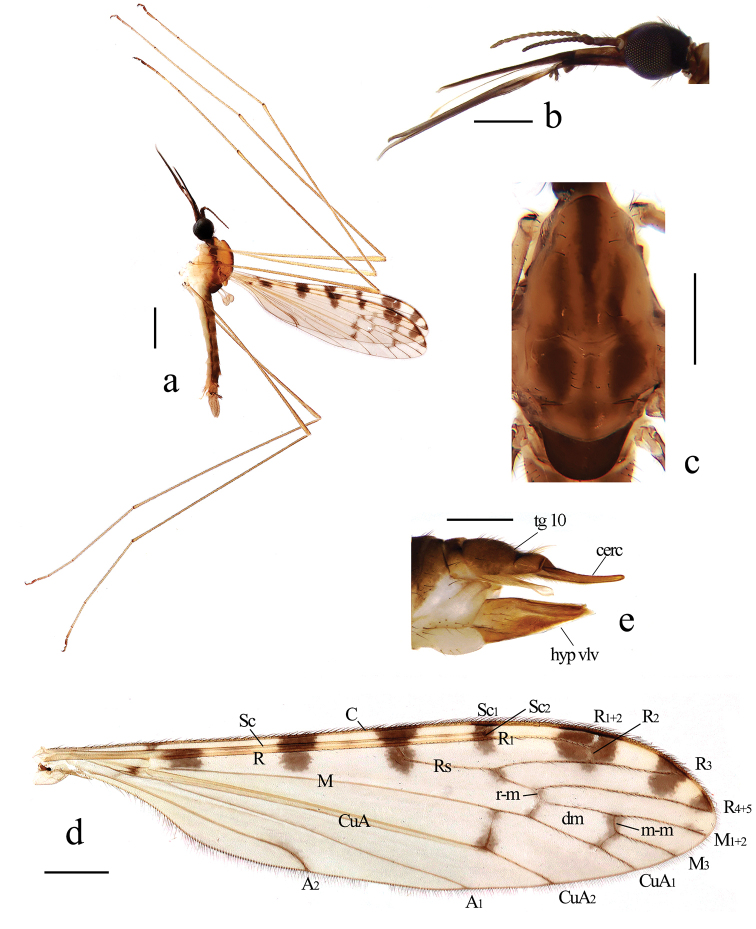
*Geranomyia jiulongensis* sp. nov. **a** Male habitus, lateral view **b** head, lateral view **c** thorax, dorsal view **d** wing **e** ovipositor, lateral view. Scale bars: 1.0 mm (**a**); 0.5 mm (**b–d**); 0.2 mm (**e**).

Head (Fig. 1b). Black. Setae on head black. Antenna length 1.2–1.3 mm, brownish black. Scape cylindrical. Pedicel nearly globose. Flagellomeres oval, terminal flagellomere with tip knob-like. Mouthparts brownish black with black setae.

Thorax (Fig. 1c). Pronotum yellow with a broad dark brown median stripe. Prescutum yellow, with three broad, dark-brown longitudinal stripes, each lateral stripe about 1/2 length of median stripe. Scutum pale yellow, with a dark-brown longitudinal stripe at middle area, each lobe with a large, dark-brown spot. Scutellum yellow, with two sides and anterior region dark brown, posterior region with a dark-brown spot. Mediotergite brownish black. Pleuron of thorax (Fig. 1a) yellow, with a broad, dark-brown stripe extending from cervical region to mediotergite. Setae on thorax brownish black. Coxae pale yellow; trochanters pale yellow; femora brownish yellow, with fore femur paler; tibiae brownish yellow; tarsi brownish yellow, with terminal three segments darker. Setae on legs brownish black. Wing (Fig. 1d) tinged pale brownish with a brownish-black pattern: seven large spots on costal region; seams along cord, m-m and basal section of M_3_; a spot at fork of Rs; a very light spot at sub-tip of A_2_. Veins brownish yellow, darker in clouded areas. Venation: Sc long, Sc_1_ ending near fork of Rs, Sc_2_ at its tip; basal section of CuA_1_ nearly its length before fork of M. Halter length 1.1–1.2 mm, pale yellow with base of stem dark brown.

Abdomen (Fig. 1a). Tergites brownish yellow with caudal halves dark brown. Sternites pale yellow. Setae on tergites brown and on sternites white.

**Figure 2.**
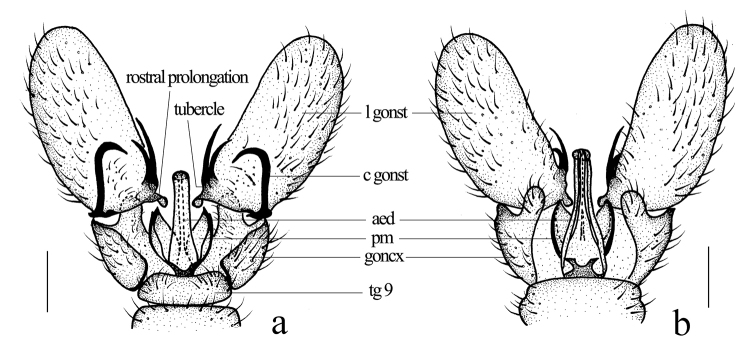
*Geranomyia jiulongensis* sp. nov. **a** male hypopygium, dorsal view **b** male hypopygium, ventral view. Scale bars: 0.2 mm.

Hypopygium (Fig. 2). Posterior margin of ninth tergite slightly emarginate. Gonocoxite slender with an elongate and blunt-apexed ventromesal lobe. Clasper of gonostylus arched at 2/3 of length, tip acute. Lobe of gonostylus large with an arched rostral prolongation armed with two basal spines arising from two tubercles. Paramere stout, wide at base, distal part trianglar. Aedeagus long, with two apical lobes.

**Female.** Body length 5.5–6.4 mm, wing length 5.0–5.8 mm, mouthparts length 2.0–2.5 mm. Similar to male, but tenth tergite (Fig. 1e) brown. Cercus brownish yellow with basal 1/2 brown. Hypogynial valve brownish yellow with tip slightly darker, tip near 2/3 way along cercus.

##### Type material.

***Holotype***: male (QAU), China: Zhejiang, Suichang, Mount Jiulong, Luohanyuan (28°23'24"N, 118°51'00"E, 517 m), 2019.VII.26, Xingyang Qian. ***Paratypes***: 10 males 5 females (QAU), same data as holotype. 1 female (QAU), China: Zhejiang, Suichang, Mount Jiulong, Longkoucun (28°18'11"N, 118°56'42"E, 305 m), 2019.VII.24, Xingyang Qian. 1 male 5 females (QAU), China: Zhejiang, Suichang, Mt. Jiulong, Xikengli (28°20'10"N, 118°55'00"E, 732 m), 2019.VII.25, Xingyang Qian. 1 male (QAU), China: Zhejiang, Suichang, Mount Jiulong, Yanping (28°22'23"N, 118°53'48"E, 667 m), 2019.VII.26, Xingyang Qian. 1 female (QAU), China: Zhejiang, Suichang, Mount Jiulong, Zuobieyuan (28°17'10"N, 118°46'42"E, 640 m), 2019.VII.28, Xingyang Qian.

##### Distribution.

China (Zhejiang).

##### Etymology.

The species is named after the type locality, Mount Jiulong.

##### Remarks.

This species is very similar to *G. radialis* but can be distinguished by the prescutum of the thorax having three broad, dark-brown stripes (Fig. 1c) and the lobe of the gonostylus being long and more than twice the length of the gonostylus clasper (Fig. 2a). In *G. radialis*, the prescutum has a narrow brown median stripe, and the lobe of the gonostylus is short and slightly exceeds the gonostylus clasper (Alexander 1930). This new species is also somewhat similar to *G. immobilis* (Alexander, 1932) from the Philippines in its pattern and wing venation but can be easily distinguished from it by the pleuron of the thorax being yellow with a broad dark brown stripe extending from the cervical region to the mediotergite (Fig. 1a) and the lobe of the gonostylus with two spines arising from two tubercles. In *G. immobilis*, the pleuron of the thorax is chiefly dark brown, with the sternopleurite light yellow, and the lobe of the gonostylus has two spines arising from a common tubercle (Alexander 1932).

#### Geranomyia subablusa sp. nov.

Taxon classificationAnimaliaDipteraLimoniidae

7C7EA098-9FEB-5EAB-B69D-96E43B903DF0

http://zoobank.org/2F4E8F84-2595-4B4C-9026-6ED380AA499D

Figures 3 , 4

##### Diagnosis.

Prescutum yellow with three broad, brown longitudinal stripes. Pleuron of thorax yellow, with a broad brown stripe. Wing with seven large spots on costal region, with second and third spots combined between C and Sc; Sc_1_ ending about 2/5 of Rs; basal section of CuA_1_ more than 2/3 of its own length before fork of M. Lobe of gonostylus large with a large rostral prolongation armed with two long, slender spines, one arising from a large fleshy tubercle, other one directly arising from rostral prolongation.

##### Description.

**Male.** Body length 6.2–6.5 mm, wing length 6.0–6.3 mm, mouthparts length 2.4–2.5 mm.

**Figure 3.**
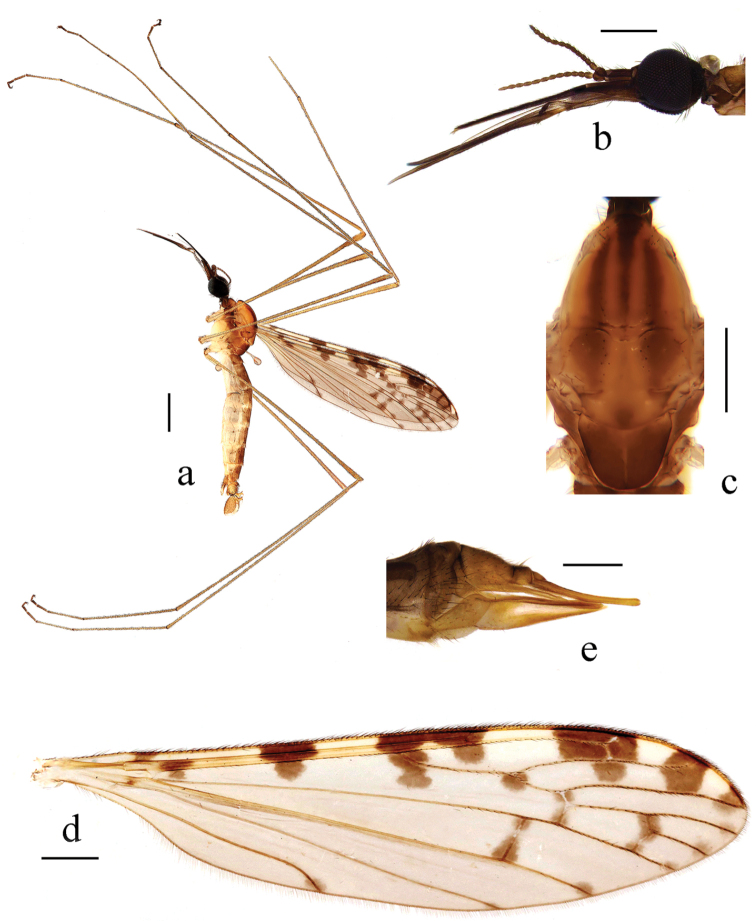
*Geranomyia subablusa* sp. nov. **a** Male habitus, lateral view **b** head, lateral view **c** thorax, dorsal view **d** wing **e** ovipositor, lateral view. Scale bars: 1.0 mm (**a**); 0.5 mm (**b–d**); 0.2 mm (**e**).

Head (Fig. 3b). Brownish black. Setae on head black. Antenna length 1.2–1.3 mm, dark brown. Scape cylindrical. Pedicel nearly globose. Flagellomeres oval, terminal flagellomere with tip knob-like. Mouthparts dark brown, with black setae.

Thorax (Fig. 3c). Pronotum brownish yellow, with a broad, dark-brown, median stripe. Prescutum yellow with three broad, brown, longitudinal stripes; each lateral stripe about 3/4 length of median stripe. Scutum pale yellow; each lobe with a large brown spot. Scutellum yellow, with brown sides; posterior region with a brown spot. Mediotergite dark brown. Pleuron of thorax (Fig. 3a) yellow, with a broad, brown stripe extending from cervical region to mediotergite. Setae on thorax brownish black. Coxae yellow; trochanters yellow; femora brownish yellow; tibiae brownish yellow; tarsi brownish yellow, with terminal three segments darker. Setae on legs brownish black. Wing (Fig. 3d) tinged with pale brownish with brownish black pattern: seven large spots on costal region, with second and third spots combined between C and Sc; seams along cord, m-m and basal section of M_3_; spots at fork of Rs and tip of M_1+2_, M_3_, CuA_1_ and A_2_; a very light and small spot at tip of A_1_. Veins brownish yellow, darker in clouded areas. Venation: Sc long, Sc_1_ ending about 2/3 of Rs, Sc_2_ at its tip; basal section of CuA_1_ more than 2/3 of its own length before fork of M. Halter length 1.1–1.2 mm, yellowish white.

Abdomen (Fig. 3a). Tergites brown. Sternites pale yellow. Setae on tergites brown and on sternites white.

**Figure 4.**
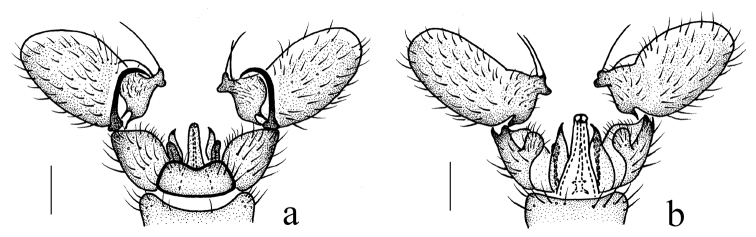
*Geranomyia subablusa* sp. nov. **a** male hypopygium, dorsal view **b** male hypopygium, ventral view. Scale bars: 0.2 mm.

Hypopygium (Fig. 4). Posterior margin of ninth tergite emarginate. Gonocoxite stout with a blunt-apexed ventromesal lobe. Clasper of gonostylus arched at 2/3 of length, tip acute. Lobe of gonostylus large, with a large rostral prolongation armed with two long, slender spines, one arising from a large fleshy tubercle at sub-tip of rostral prolongation, other one directly arising from middle of rostral prolongation. Paramere slender, wide at base, distal part triangular. Aedeagus relatively long, with two apical lobes.

**Female.** Body length 6.0–7.0 mm, wing length 6.0–6.5 mm, mouthparts length 2.3–2.5 mm. Similar to male, but tenth tergite (Fig. 3e) brown, with tip brownish yellow. Cercus brownish yellow, with basal 1/2 brown, long. Hypogynial valve brownish yellow, slender, and long, with tip near 2/3 way along cercus.

##### Type material.

***Holotype***: male (QAU), China: Zhejiang, Suichang, Mount Jiulong, Luohanyuan (28°23'24"N, 118°51'00"E, 517 m), 2019.VII.26, Xingyang Qian. ***Paratypes***: 4 males 10 females (QAU), same data as holotype. 2 males 2 females (QAU), China: Zhejiang, Suichang, Mount Jiulong, Longkoucun (28°18'11"N, 118°56'42"E, 305 m), 2019.VII.24, Xingyang Qian.

##### Distribution.

China (Zhejiang).

##### Etymology.

The name of the new species refers to the *G. ablusa* (Alexander, 1967), as the two species are very similar morphologically.

##### Remarks.

This species is very similar to *G. ablusa* from India but can be distinguished from it by the femora being uniformly brownish yellow (Fig. 3a), the yellowish white halter, and the aedeagus lacking genital openings near the apical lobes (Fig. 4b). In *G. ablusa*, the femora have vague, pale brown, subterminal rings, the halter is dark brown, and the aedeagus has the genital openings subterminal and lateral in position to the apical lobes (Alexander 1967c).

#### Geranomyia nigra

Taxon classificationAnimaliaDipteraLimoniidae

Zhang, Zhang & Yang, 2016

DC03BB46-C0AF-54F2-93E1-15F4F6EAEC0A

Geranomyia nigra : Zhang et al. 2016: 150. Type locality: Fuxing, Taoyuan, Taiwan (China).

##### Specimens examined.

***Holotype***: male (CAU), China: Taiwan, Taoyuan, Fuxing (24°48'36"N, 121°20'55"E, 420 m), 2013.VI.10, Wenliang Li (light trap). ***Other material***: 2 males 2 females (QAU), China: Zhejiang, Suichang, Mount Jiulong, Luohanyuan (28°23'24"N, 118°51'00"E, 517 m), 2019.VII.26, Xingyang Qian. 1 male 2 females (QAU), China: Zhejiang, Suichang, Mount Jiulong, Longkoucun (28°18'11"N, 118°56'42"E, 305 m), 2019.VII.24, Xingyang Qian.

##### Distribution.

China (Gansu, Sichuan, Yunnan, Zhejiang, Guangxi, Taiwan).

#### Geranomyia suensoniana

Taxon classificationAnimaliaDipteraLimoniidae

(Alexander, 1929)

23C26147-500C-5ACB-9987-B5EA9A509F0F

Limonia (Geranomyia) suensoniana : Alexander 1929a: 330. Type locality: hills south of Ningbo, Zhejiang (China).

##### Specimens examined.

***Holotype***: male (USNM), China: Zhejiang, hills south of Ningbo, 1925.V.1, E. Suenson. ***Other material***: 2 male 4 females (QAU), China: Zhejiang, Suichang, Mount Jiulong, Luohanyuan (28°23'24"N, 118°51'00"E, 517 m), 2019.VII.26, Xingyang Qian.

##### Distribution.

China (Zhejiang).

## Supplementary Material

XML Treatment for Geranomyia jiulongensis

XML Treatment for Geranomyia subablusa

XML Treatment for Geranomyia nigra

XML Treatment for Geranomyia suensoniana
